# Measuring Relationship Influences on Romantic Couples’ Cancer-Related Behaviors During the COVID-19 Pandemic: Protocol for a Longitudinal Online Study of Dyads and Cancer Survivors

**DOI:** 10.2196/48516

**Published:** 2024-07-31

**Authors:** Jennifer M Bowers, Chloe O Huelsnitz, Laura A Dwyer, Laurel P Gibson, Tanya Agurs-Collins, Rebecca A Ferrer, Amanda M Acevedo

**Affiliations:** 1 Behavioral Research Program National Cancer Institute National Institutes of Health Rockville, MD United States; 2 Cape Fox Facilities Services Manassas, VA United States; 3 Department of Psychology and Neuroscience University of Colorado Boulder Boulder, CO United States

**Keywords:** cancer prevention, COVID-19, risk perceptions, dyads, romantic relationships, cancer, oncology, survivor, survivors, dyad, spouse, spousal, partner, health behavior, health behaviors, cohabiting, cohabit, study design, recruit, recruitment, methodology, methods, enrol, enrolment, enroll, enrollment

## Abstract

**Background:**

Research has established the effects of romantic relationships on individuals’ morbidity and mortality. However, the interplay between relationship functioning, affective processes, and health behaviors has been relatively understudied. During the COVID-19 pandemic, relational processes may influence novel health behaviors such as social distancing and masking.

**Objective:**

We describe the design, recruitment, and methods of the relationships, risk perceptions, and cancer-related behaviors during the COVID-19 pandemic study. This study was developed to understand how relational and affective processes influence romantic partners’ engagement in cancer prevention behaviors as well as health behaviors introduced or exacerbated by the COVID-19 pandemic.

**Methods:**

The relationships, risk perceptions, and cancer-related behaviors during the COVID-19 pandemic study used online survey methods to recruit and enroll 2 cohorts of individuals involved in cohabiting romantic relationships, including 1 cohort of dyads (n=223) and 1 cohort of cancer survivors (n=443). Survey assessments were completed over 2 time points that were 5.57 (SD 3.14) weeks apart on average. Health behaviors assessed included COVID-19 vaccination and social distancing, physical activity, diet, sleep, alcohol use, and smoking behavior. We also examined relationship factors, psychological distress, and household chaos.

**Results:**

Data collection occurred between October 2021 and August 2022. During that time, a total of 926 participants were enrolled, of which about two-thirds were from the United Kingdom (n=622, 67.8%) and one-third were from the United States (n=296, 32.2%); about two-thirds were married (n=608, 66.2%) and one-third were members of unmarried couples (n=294, 32%). In cohorts 1 and 2, the mean age was about 34 and 50, respectively. Out of 478 participants in cohort 1, 19 (4%) identified as Hispanic or Latino/a, 79 (17%) as non-Hispanic Asian, 40 (9%) as non-Hispanic Black or African American, and 306 (64%) as non-Hispanic White; 62 (13%) participants identified their sexual orientation as bisexual or pansexual, 359 (75.1%) as heterosexual or straight, and 53 (11%) as gay or lesbian. In cohort 2, out of 440 participants, 13 (3%) identified as Hispanic or Latino/a, 8 (2%) as non-Hispanic Asian, 5 (1%) as non-Hispanic Black or African American, and 398 (90.5%) as non-Hispanic White; 41 (9%) participants identified their sexual orientation as bisexual or pansexual, 384 (87.3%) as heterosexual or straight, and 13 (3%) as gay or lesbian. The overall enrollment rate for individuals was 66.14% and the overall completion rate was 80.08%.

**Conclusions:**

We discuss best practices for collecting online survey data for studies examining relationships and health, challenges related to the COVID-19 pandemic, recruitment of underrepresented populations, and enrollment of dyads. Recommendations include conducting pilot studies, allowing for extra time in the data collection timeline for marginalized or underserved populations, surplus screening to account for expected attrition within dyads, as well as planning dyad-specific data quality checks.

**International Registered Report Identifier (IRRID):**

DERR1-10.2196/48516

## Introduction

Research has shown that romantic relationships can impact individuals’ morbidity and mortality outcomes; however, there is a need to understand how partners in romantic relationships influence one another’s health outcomes, including through their health behaviors [1]. In the context of cancer prevention and control, most of the research has focused on the role of romantic partners at the stages of diagnosis, treatment, and survivorship (eg, as caregivers), rather than the role of partners at the stages of prevention and detection [2,3]. Romantic relationships often involve mutual and enduring influence over time, shared decision-making regarding cancer-relevant health behaviors (eg, diet), and shared social and living environments. Therefore, focusing cancer prevention efforts on individuals rather than on romantic dyads, when feasible, may represent a missed opportunity for positive impact. As such, there is a need to understand how relational processes in romantic relationships influence cancer prevention. In this study, we describe the protocol of the relationships, risk perceptions, and cancer-related behaviors during the COVID-19 pandemic (R2C2) study, which aimed to (1) examine ways in which romantic partners influence each other across a wide range of cancer prevention behaviors and COVID-19–related behaviors, (2) examine ways in which the COVID-19 pandemic has influenced both cancer control and romantic relationships, and (3) recruit participants from historically underrepresented and medically underserved backgrounds.

The COVID-19 pandemic has disrupted many stages along the cancer control continuum including the health behaviors individuals engage in, their ability to receive treatment, survivorship care, and even the grieving process [4-8]. In addition, the pandemic has disrupted social relationships [9,10], an important social factor contributing to cancer prevention and control, both in terms of changing the ways in which people interact and by introducing new stressors and opportunities for conflict [11]. The COVID-19 pandemic affords the opportunity to examine not only disruptions across the cancer prevention and control continuum and disruptions to individuals’ romantic relationships but also to study ways in which romantic partners influence each other’s health. Thus, we sought to examine ways in which cancer prevention and control have been disrupted during the pandemic, how romantic relationship functioning has been disrupted, and how romantic partners influence each other’s COVID-19–related behaviors, such as social distancing, masking, and vaccination.

The COVID-19 pandemic has also widened gender, sexual orientation, and racial or ethnic disparities in health and well-being [12-15]; yet, very little is known about how close relationships during the pandemic might influence the health behaviors of these populations. Specifically, dyadic health research often fails to include people with racialized identities as well as people who identify with lesbian, gay, bisexual or pansexual orientations (LGB), and who can be characterized as underrepresented populations in the literature. In addition, cancer survivors have been particularly vulnerable to COVID-19 during the pandemic [16]. The greater risk of health consequences for cancer survivors due to the COVID-19 pandemic, combined with evidence gaps in the existing survivorship literature [17], demonstrates a critical need to better understand the mechanisms of health behavior and risk perceptions during survivorship. Thus, a multilevel analysis of health behaviors, including measuring the role of relationships is needed to fill these evidence gaps. Close relationships during the pandemic may impact health behaviors differently across the cancer control continuum and intersect by identity. Therefore, we aimed to recruit cancer survivors and underrepresented populations.

Dyadic research, in which both members of a dyad (eg, a romantic couple) are assessed on the same or similar measures, is an important methodology that allows for a better understanding of how individuals in a relationship influence one another’s health. Dyadic methods can estimate the degree of correspondence (ie, similarity) in health behaviors and behavioral correlates between 2 members of a dyad. These analyses also provide insights into how individuals’ behaviors are influenced by their partners in a way that cannot be accomplished by models that assume participants are independent of one another.

Research designs that investigate the intricate interplay of environmental factors, health behavior patterns, and shared risk perceptions within dyadic relationships, particularly romantic partnerships, are valuable for a better understanding of individuals’ health. Numerous studies have shown that close relationship partners, and the quality of those relationships, have significant impacts on mortality, morbidity, and their underlying determinants [18-20]. Furthermore, recent research focusing on romantic relationships during the COVID-19 pandemic has highlighted the profound impact of mental health indicators related to COVID-19, such as psychological stress and depressive symptoms, on the functioning of these relationships [11,21]. In addition, changes in certain health behaviors during the pandemic, such as increased alcohol use, have been associated with heightened COVID-19–related relationship conflict [22].

Understanding the specific dynamics of health-related behaviors and perceptions within romantic partnerships, especially given the unique challenges posed by events like the COVID-19 pandemic, is crucial. The pandemic introduced stressors and changes that influence relationship processes, which in turn may influence how couples engage in health or health-risk behaviors. Investigating these dynamics can provide insights into how environmental factors, health behaviors, and shared risk perceptions influence the health and well-being of both individuals and their relationships.

Recognizing the largely untapped potential, as well as the gaps and shortcomings in the existing literature such as homogenous (ie, White and heterosexual) samples and frequent use of cross-sectional approaches, led to the conceptualization of this study examining the complex interplay of multilevel factors in this context, including relationship variables, risk perceptions, cancer-related behaviors, and COVID-19–related factors, as well as structural and environmental factors that may play a role.

**Figure 1:**
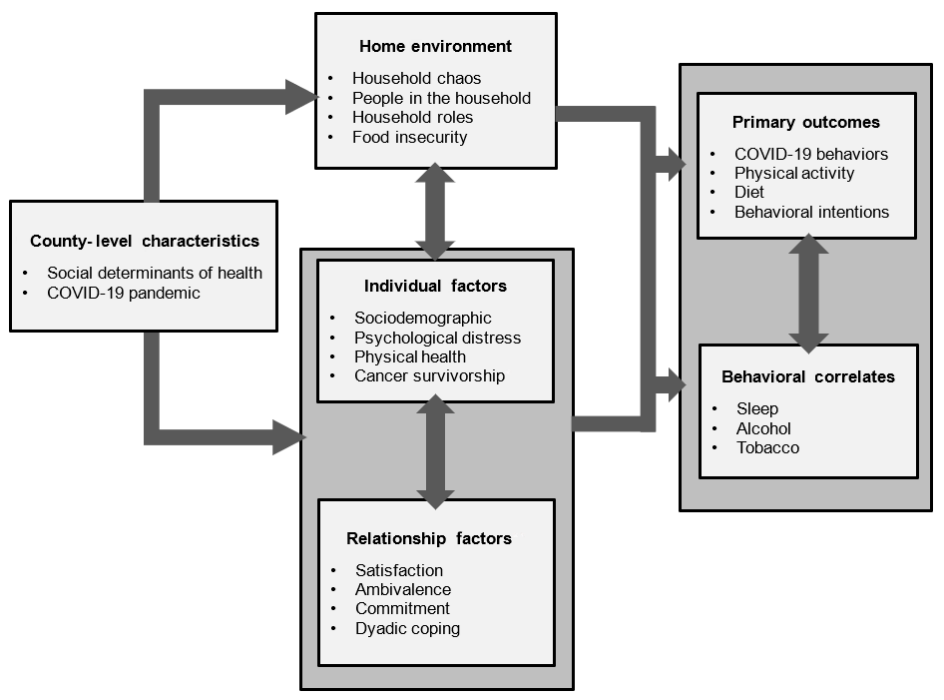
A conceptual framework for the relationships, risk perceptions, and cancer-related behaviors during the COVID-19 pandemic (R2C2) study of cancer survivors and romantic dyads.

To address evidence gaps in the literature and to assess how the ongoing COVID-19 pandemic may influence dyadic processes relevant to health, we designed a dual-cohort repeated-measures online survey study for the development and testing of comprehensive models that identify dyadic influences on cancer risk and prevention behaviors. The first cohort in R2C2 consisted of cohabitating, romantic dyads while the second cohort consisted of individual cancer survivors who cohabitated with a romantic partner. R2C2 included the measurement of multiple relationship variables as well as multiple health-related outcomes and moderators as a reflection of a more complex health behavior theoretical framework (discussed in the “Study Development” section; Figure 1). Surveys were developed with the intention to include analysis of the effects of individual differences, emotion coregulation, health behaviors, COVID-19 and cancer-related risk perceptions, and contextual or environmental factors.

The purpose of this methods report is to describe methodologies and approaches that are seldom described in detail but will be valuable for progressing future research on dyadic relationships and health. There is limited detailed research on best practices for recruiting specific samples, such as romantic dyads and cancer survivors, through online panels. Furthermore, dyadic research is complex and challenging (eg, deciding on whether to prioritize dyadic completion as a recruitment strategy). Thus, this report aims to (1) detail the methodological decisions, tools, and processes that were used during the R2C2 study, (2) report recruitment, enrollment, and response data, and (3) reflect on best practices and limitations to guide future study design.

## Methods

### Study Development

We engaged in a multipronged approach to develop the study in a way that would address the abovementioned gaps in cancer prevention research among dyads. First, we identified that other federally sponsored surveys did not already focus on multiple preventive health behaviors among romantic couples. Second, we conducted a series of literature searches in which we scanned published literature on specific cancer prevention behaviors to (1) identify the scope of previous dyadic research that included outcomes of diet, physical activity, sleep, tobacco use, and alcohol use and (2) examine measures and methods used in previous studies of romantic relationships and social support. Third, we conducted a portfolio analysis to determine National Cancer Institute (NCI)–funded grants that involved dyads. We identified the types of dyads sampled, the purpose and specific aims, outcomes, and the constructs and measures used in these studies. Fourth, we engaged in a survey development process in which we asked experts to identify and refine our survey measures. We identified NCI experts on relationships and health and held retreats with them to identify key areas and additional external experts (some of whom were identified during the literature searches) who could provide feedback on our conceptual framework (Figure 1), constructs, and measures. This process was iterative, such that we sent the conceptual framework to experts for feedback, collated that feedback to refine the areas, had NCI fellows conduct reviews to identify relevant items in published work and existing surveys, and sent a long-form survey to the experts for comment and reductions or additions. We also solicited feedback on dyadic research gaps from the general scientific community and the public through the NCI Behavioral Research Program website before selecting the items included in the survey.

These efforts helped refine the methods and content of the study. For example, through this feedback, we added an assessment of stressful life events commonly experienced among adults to examine how these events influence individuals’ and dyads’ cancer risk behaviors. We also collected data at more than one time point to meaningfully study social exchange and support processes within couples. Our conversations with other researchers also confirmed the importance of recruiting dyads that included individuals who have been underrepresented in the literature (ie, individuals with racialized identities and individuals who identify with LGB sexual orientations). We maintained a focus on these recommendations when deciding how to incorporate pandemic-related influences into the survey.

### Recruitment and Enrollment

We recruited 2 cohorts of participants using Prolific, a UK-based online platform for research participant pool recruitment and paid participation [23]. Prolific has protocols intended to fairly compensate participants and deliver high-quality data to researchers. At the time of our study recruitment, Prolific had a pool of approximately 122,110 total available users, all of whom were 18 years or older. We used their standard sample recruitment method and prescreening tool to launch our studies. The prescreening tool allows the recruitment of selected groups of participants based on an extensive set of screening questions carried out by Prolific for each user. Screening criteria can be very specific to meet the needs of a study; for example, researchers can select participants who report a diagnosis of a specific chronic illness, like cancer. Multiple types of screening criteria can be applied, and researchers can preview an estimate of the available user pool for their specific criteria when they create a new study. This estimate is based on the number of potential participants who meet the selected criteria and who have been active on Prolific in the past 90 days.

**Figure 2:**
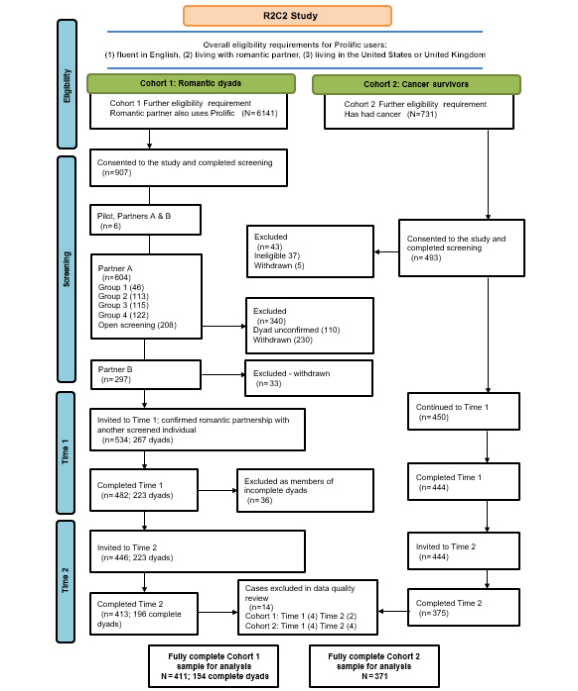
Relationships, risk perceptions, and cancer-related behaviors during the COVID-19 pandemic (R2C2) study flow diagram.

Eligibility criteria used for both cohorts of adults (cohort 1 of romantic partners and cohort 2 of cancer survivors) were selected using the prescreening tool and included: (1) fluent in English, (2) living with a spouse or partner, and (3) living in the United States or the United Kingdom. In addition, cohort 1 required both partners to affirm using Prolific’s prescreening tool that they have a romantic partner who also has a Prolific account, and cohort 2 required individuals to report having had cancer using Prolific’s prescreening tool (cohort-specific considerations are further discussed in this paper). At the time of initiation of data collection, according to Prolific, the resulting number of participants eligible for the studies was 6141 for cohort 1 and 731 for cohort 2. Figure 2 contains details regarding study recruitment.

### Stratification

Prolific is typically found to have a more diverse participant pool and higher quality data compared with other online recruitment platforms such as Amazon Mechanical Turk [23,24]. However, to ensure adequate recruitment of individuals from underrepresented populations, we implemented a stratification approach for our study. While we initially intended to use this stratification approach for both cohorts, the small number of eligible cancer survivors from diverse backgrounds necessitated opening recruitment to the entire pool of cancer survivors to achieve adequate statistical power. Therefore, the following stratification method was only used throughout the entire study for cohort 1. For cohort 2, the stratification method was implemented toward the end of data collection to attempt to better balance race or ethnicity in the sample.

Prolific offers a built-in recruitment process to balance across sex but balancing across other demographic groups requires researchers to create multiple copies or versions of their study to select the specified prescreening criteria for each. For cohort 1, we created 4 versions of the study (all linked to the same single Qualtrics survey) to meet recruitment goals for the individuals, identifying as (1) people with both a racialized identity and an LGB sexual orientation (group 1), (2) people with a racialized identity and heterosexual orientation (group 2), people who identified as White and an LGB sexual orientation (group 3), and (3) people who identified as White and heterosexual (group 4). Specifically, Prolific-defined categories for “ethnicity (simplified)” and “sexual orientation” were used so that individuals who selected the Black, Asian, mixed, and other response options represented people with underrepresented racialized identities and individuals who selected the homosexual, bisexual, asexual, and other response options represented people with underrepresented sexual orientations, respectively. Partner A’s Prolific screening information was used to assign dyads to a stratification group.

Finally, to confirm that eligibility criteria data provided to Prolific by users remained accurate at the time of enrollment (eg, still living with a romantic partner) we conducted a brief validation of prescreening data by readministering screening questions used by Prolific before administering “time 1” surveys to each cohort. Enrollment in study screening was ongoing for several months of the iterative data collection process. Every 48 hours, Prolific sends email invitations to a random proportion of users who are eligible for studies that have not reached their maximum number of submissions [25].

### Data Collection

Cohort 1 data collection ran from October 13, 2021, to June 10, 2022, and cohort 2 from December 20, 2021, to August 31, 2022. Surveys were administered online using Qualtrics. Questionnaires and items used in each of the surveys were similar across the 2 cohorts and the 2 time points, and summaries of these measures (constructs and example items) can be found in Multimedia Appendix 1.

Demographic characteristics were only assessed at time 1. Prolific and Qualtrics have integration features available to ensure continuity between participants’ completing studies on Qualtrics and receiving completion credit on Prolific. We used these integration features to automatically record participants’ Prolific IDs in the embedded Qualtrics data, as well as provided a completion code to participants at the end of their Qualtrics survey to enable their receipt of credit on the Prolific platform. For further information about this process, we recommend visiting Prolific’s research help center [25]. After building our survey in Qualtrics by inserting our Institutional Review Board–approved questionnaires, we estimated the time for time 1 and time 2 completion to be about 20-30 minutes. Our study team then piloted the survey to confirm the formatting was displaying as intended and the completion time estimates were correct. Accordingly, we set the Prolific time effort as 30 minutes. The screening assessment for cohort 2 was built-in to the beginning of the time 1 survey, and access to the survey was contingent on this screening.

Before launching the surveys, another phase of pilot testing was done. After study team members piloted the surveys to confirm format and timing, 6 Prolific users who met the criteria for cohort 1 (ie, 3 dyads) were invited as pilot participants. These participants helped us test the process of screening, tracking, matching, inviting, and compensating eligible dyads in Prolific and Qualtrics. In addition, 5 of these participants took part and provided proof of concept for the study setup. Because changes were not made to the study following these pilot tests, the 5 pilot participants were included in the final data set for cohort 1.

Each participant who successfully completed time 1 was compensated for their participation within 2 weeks. After completing time 1, invitations to complete time 2 for additional compensation were sent to eligible individuals after 4 weeks, which was done by pasting each time 1 participant’s Prolific ID into a “custom-allow list” for each of the cohort’s respective time 2 surveys on Prolific.

### Considerations for Cohort 1

Prolific enables dyadic data collection from romantic couples [25]. The key feature used to achieve this is the prescreening question, “Do you have a romantic partner who has a Prolific account and are you willing to participate in studies as a couple?” It is required to create 2 versions of a study to recruit cohabitating couples on Prolific due to a mechanism that blocks duplicate IP addresses from participating in the same study. Therefore, our screening process entailed administering 4 versions for Partner A (discussed in “Stratification” section), and 1 additional survey for Partner B. The label of Partner A was assigned to the first user in a dyad who completed our screening process. In addition to confirming eligibility criteria during screening, we also asked Partner A to provide the Prolific ID of their romantic partner so that we could add them to the allowed list for the Partner B version of the study. Finally, demographic information from Partner A was used to assign dyads to stratification groups. Once we obtained screening information for Partner A, a unique Dyad ID was assigned.

Once Partner B completed the screening and confirmed the Prolific ID of their romantic partner, we invited each partner to enroll in the respective A and B copies of the time 1 survey. These survey copies were identical, with the same Qualtrics link, and existed as a workaround to the IP address blocking mechanism. For time 2, invitations to complete the survey were only sent to dyads 4 weeks after both partners had completed time 1 (ie, 4 weeks after the second partner completed time 1. Both partners’ time 1 completion was a requirement for time 2 eligibility). In addition, to minimize excessive time elapsing between the time points, we sent reminder messages through private messages after about 8 weeks to the participants for whom their partner had already completed time 1.

### Considerations for Cohort 2

The unique aspect of the data collection process for cohort 2, the cancer survivor cohort, required screening individuals for any history of cancer. The Prolific prescreening question used for this was, “Do you have–or have you had–any condition, injury, or chronic illness?” and the response required for eligibility was “Cancers.” In the time 1 survey, we asked participants to indicate their specific cancer type from a list we provided. In cases where a cancer type was not listed, we accepted this information through secure Prolific messages from participants. All other data collection processes followed according to the description in the section, “Data Collection.” Cohort 2 was not dyadic; we only required participants to report cohabitating with their romantic partner.

We were unable to target cohort 2 using the stratified enrollment strategy we used for cohort 1 due to the small sample size of cancer survivors participating on Prolific. After recruiting cohort 2 for 8 months, we examined the race or ethnicity of the sample and decided to try to focus recruitment on participants who were from underrepresented racialized backgrounds. This resulted in the recruitment of 7 more participants to the cancer survivor cohort.

### Ethical Considerations

After a human subject ethics review, the study was classified as exempt by the National Institutes of Health Office of Institutional Review Board Operations (#000535), under the exemption for “uses educational tests, surveys, interviews, or observations of public behavior”. Each participant provided informed consent online (with the choice to instead opt out) before beginning the study. Per Prolific rules, participants were not asked to provide any personally identifying information. The research team has access to participants’ Prolific ID, but this ID can only be linked to their participants’ personally identifying information by Prolific. Prolific staff members do not have access to any of the study data, which were collected using Qualtrics. As such, no individual or entity is able to connect participants’ personally identifying information with their study data. Compensation was set on Prolific as US $3.50 for time 1 and US $5 for time 2. The increased incentive was intended to reduce attrition and incentivize participation at follow-up [26]. In addition, to properly screen each member of the dyads in cohort 1, US $0.25 was awarded for the separate screening process.

### Data Quality and Analysis

A power analysis to determine the number of participants to enroll for cohort 1 was calculated using the online tool, Power Analysis for the Actor-Partner Interdependence Model (APIMPowerR) [27] using the following assumptions: α=.05, 85% power, moderate effect size for actor pathways (Cohen *d*=0.50), small effect size for partner pathways (Cohen *d*=0.30), *r*=0.25 correlation of actor with partner variables, and *r*=0.25 correlation of errors. These analyses indicated 201 dyads (n=402 participants) would yield adequate power to detect partner pathways (and 74 dyads, or n=148 participants, would yield adequate power to detect actor pathways). For cohort 2, using G*Power [28], we conducted power analyses for multiple linear regressions using assumptions such as α=.05, 85% power, small-medium effect size (f^2^=0.05), and up to 10 predictors. This analysis indicated that 372 participants would yield adequate power to detect an *R*^2^ deviation from 0. The power analysis for each cohort helped inform the minimum number of participants to recruit.

We implemented several measures to promote high-quality final data across both cohorts. In building the study surveys in Qualtrics, we included CAPTCHA (Completely Automated Public Turing test to tell Computers and Humans Apart) technology that detects bot-like responses. When the data became available, we checked for abnormally fast response rates, reviewed responses to our attention check item (Multimedia Appendix 1), and reviewed couple concordance on expected responses in cohort 1 (eg, county of residence). To identify fast responses to the surveys, we set a parameter of 2 SDs under the mean completion time, identifying no responses to remove on this basis. Among the few participants who had failed the attention check item, we reviewed their other responses to identify strings of low-quality data (eg, straight-lining), removing no respondents on this basis; rather, we added a flag variable to the data set to alert analysts of failed attention checks. We contacted Prolific to verify that flagged users were in good standing, and with their recommendation, we removed 2 cases from the cohort 1 data sets that we flagged for suspicious discordance in their data. Another individual was removed from cohort 1 time 1 after a review of their survey responses indicating they were not based in the United States or the United Kingdom. Furthermore, 1 more case was removed for being a duplicate. For cohort 2, 1 case was removed from time 1 for being a duplicate, and 1 case was removed from time 2 for having incomplete data. Finally, a check for participants who may have entered both cohorts (despite attempts to exclude based on previous participation—ongoing studies were not able to be factored into these exclusions) revealed 3 cases that were removed from each time point in cohort 2 (Figure 1).

Enrollment is defined in our study in the following way: enrolled users at each time point refer to the number who met all the criteria (and were therefore paid for their participation), that are (1) were found to be eligible through our screening process, (2) completed the survey on Qualtrics, (3) did not withdraw their participation on Prolific, and (4) did not time out on Prolific after the 87 minutes allowed (calculated by Prolific based on estimated completion time of 30 minutes).

## Results

### Enrollment

The overall enrollment rate for the R2C2 study was 66.14%, with 926 individuals enrolled in time 1 after screening 1400. The overall completion rate for those who completed both time points out of those who were invited to time 1 was 80.08%. Enrollment data for each cohort and time point are reported below and in Figure 1.

### Cohort 1 Enrollment

#### Screened Participants

We screened 907 individuals; that is, 907 individuals consented to the study and completed the screener (and were compensated). This number includes pilot participants. This number does not include participants who returned their screener or who timed out of the screener (ie, who did not complete the screener).

#### Invited Participants

Of the 907 individuals who completed the screener, we obtained 267 dyads (534 individuals). That is, of the 907 individuals who completed the screener, 534 (58.9%) individuals confirmed their romantic partnership with another screened individual, resulting in 267 dyads being invited to take the time 1 survey. Individuals were only sent the time 1 survey if they were confirmed to be in a relationship with another person who completed the screener.

#### Time 1 Enrollment

Of the 267 dyads (534 individuals) who were sent the time 1 survey, 482 (90.3%) individuals and 223 (83.5%) dyads had fully completed time 1 surveys. Individuals were only sent the time 2 survey if they and their partner both completed the time 1 survey.

#### Time 2 Enrollment

Of the 446 individuals (making up the 223 dyads) who were sent the time 2 survey, 413 (92.6%) individuals and 196 (87.9%) dyads completed the time 2 survey.

#### Dyadic Enrollment Rate

In sum, screening 907 individuals resulted in complete dyadic data for 196 dyads (392, 43.2% individuals). In other words, 43.2% of the screened individuals confirmed they were in a dyad, we sent them and their partner the time 1 survey and they both completed it, and we sent them and their partner the time 2 survey 4 weeks later and they both completed it. Of the eligible dyads who were invited to the time 1 survey, 73.4% (196/267) fully completed the study.

#### Sample Characteristics

Sample characteristics were calculated based on the number of participants who completed the time 1 survey, after exclusions (discussed in the “Data Quality and Analysis” section; n=478). The mean age of participants was 34.31 (SD 9.28) years. Out of the total, 78% (373/478) were based in the United Kingdom and 22% (105/478) in the United States. The average length of participants’ romantic relationships was 10.05 (SD 7.59) years. Other characteristics, including gender, race, ethnicity, sexual orientation, history of COVID-19 infection, education, and relationship type appear in Table 1.

**Table 1:** Sample characteristics.

Characteristics			Cohort 1 (N=478)		Cohort 2 (N=440)
**Country of residence, n (%)**					
	United States	105 (22)		191 (43.4)	
	United Kingdom	373 (78)		249 (56.6)	
**Gender, n (%)**					
	Men	215 (45)		128 (29.1)	
	Women	259 (54.2)		310 (70.5)	
	Nonbinary or gender-queer	4 (<1)		2 (<1)	
Age (years), mean (SD)			34.31 (9.28)		50.25 (13.85)
**Race or ethnicity, n (%)**					
	Hispanic or Latino/a	19 (4)		13 (3)	
	Non-Hispanic American Indian or Alaska Native	0 (0)		1 (<1)	
	Non-Hispanic Asian	79 (17)		8 (2)	
	Non-Hispanic Black or African American	40 (8)		5 (1)	
	Non-Hispanic Middle Eastern or North African	2 (<1)		2 (<1)	
	Non-Hispanic multiple races selected	23 (5)		13 (3)	
	Non-Hispanic other^a^	8 (2)		0 (0)	
	Non-Hispanic White	306 (64)		398 (90.5)	
	Not reported	1 (<1)		0 (0)	
**Sexual orientation, n (%)**					
	Bisexual or pansexual	62 (13)		41 (9)	
	Heterosexual or straight	359 (75.1)		384 (87.3)	
	Homosexual, gay, or lesbian	53 (11)		13 (3)	
	Something else	4 (<1)		2 (<1)	
**History of COVID-19 infection, n (%)**					
	Yes	106 (22.2)		93 (21)	
	No	367 (76.8)		346 (78.6)	
	I don’t know	5 (1)		1 (<1)	
**Cancer type^b^, n (%)**					
	Breast	1 (<1)		138 (31.4)	
	Cervical	0 (0)		37 (8)	
	Colorectal	0 (0)		24 (5)	
	Endometrial	2 (<1)		19 (4)	
	Head and neck	0 (0)		39 (9)	
	Hematologic	3 (<1)		60 (14)	
	Lung	0 (0)		6 (1)	
	Melanoma	1 (<1)		26 (6)	
	Ovarian	0 (0)		20 (5)	
	Prostate	1 (<1)		29 (7)	
	Renal	0 (0)		12 (3)	
	Other^c^	1 (<1)		74 (17)	
	No current or past cancers	469 (98.1)		0 (0)	
**Relationship type, n (%)**					
	Married	271 (54.1)		337 (76.6)	
	Divorced	3 (<1)		10 (2)	
	Widowed	0 (0)		3 (<1)	
	Separated	2 (<1)		3 (<1)	
	Never married	25 (5)		17 (4)	
	A member of an unmarried couple	200 (39.9)		94 (21)	
**Education, n (%)**					
	Less than a high-school degree	11 (2.3)		15 (3)	
	A high-school degree or GED^d^	67 (14)		60 (14)	
	Some college but not a college degree	108 (22.6)		122 (27.7)	
	A 4-year college degree or higher	292 (61.1)		243 (55.2)	

^a^Non-Hispanic Other: participants who did not select Hispanic for ethnicity, selected “Some other race,” and did not select anything else listed in the response options for race. For cohort 1, most individuals who selected this either chose not to specify or identified with a country or region of origin (eg, “N/A,” “Caribbean,” “Moroccan,” and “Polish”).

^b^Values and percentages may sum >cohort N as they are “check all that apply” items.

^c^Other: for cohort 1, nonmelanoma skin cancer (n=1); for cohort 2, nonmelanoma skin cancer (n=36), bladder (n=9), bone (n=9), liver (n=3), oral (n=1), pancreatic (n=1), pharyngeal (n=1), and another cancer not listed (n=14).

^d^GED: General Educational Diploma.

### Cohort 2 Enrollment

#### Screened Participants

We screened 493 individuals; that is, 493 individuals consented to the study and completed the screener. Of the 493 individuals who completed the screener, 450 (91.3%) individuals were confirmed as eligible and were immediately directed to continue to time 1 if they wished.

#### Time 1 Enrollment

Of the 450 individuals who were administered the time 1 survey, 444 (98.7%) individuals completed it.

#### Time 2 Enrollment

Of the 444 individuals who were sent the time 2 survey, 375 (84.5%) individuals completed it.

#### Sample Characteristics

Sample characteristics were calculated based on the number of participants who completed the time 1 survey, after exclusions (n=440). The mean age of participants was 50.25 (SD 13.85) years. In total, 57% (249/440) were based in the United Kingdom and 43% (191/440) in the United States. The average length of participants’ romantic relationships was 21.86 (SD 14.37) years. Complete sample characteristics appear in Table 1.

## Discussion

### Principal Findings

As research across multiple disciplines has increasingly used online techniques [29], the importance of ensuring high enrollment rates, minimizing attrition, and maintaining high data quality for online studies with specific recruitment criteria and complex methods remains important. The experience of conducting the R2C2 study, which included a dyadic cohort and a cohort of cancer survivors who were surveyed across 2 waves online, can be used to inform recommendations for researchers aiming to conduct surveys using similar methodologies in online recruitment and data collection platforms. We have discussed several lessons learned from the R2C2 study and provided concrete recommendations for researchers who study romantic relationships or cancer survivorship throughout the “Discussion” section.

### Challenges and Successes During Recruitment and Enrollment

We had several objectives with respect to recruitment. First, we aimed to recruit and retain a sample of 201 dyads for cohort 1, and second, to maximize diversity in cohort 1 with respect to sexual orientation, race, and ethnicity. Third, we aimed to recruit a sample size of 372 individuals to cohort 2, and fourth, to maximize sample diversity in cohort 2 with respect to racial or ethnic background.

Despite having a relatively long data collection period of 9 months, increasing incentives for survey completion, and screening and recruiting additional individuals, we fell short of our goal of 201 dyads with complete data (final n=194 dyads with complete, quality-checked data). Our initial assumptions, using Prolific’s prescreening tool, were that 20% of individuals screened would be ineligible for participation. However, about half of the participants or their partners did not meet the inclusion criteria, or individuals’ partners did not complete the screening survey to complete the dyad. We, therefore, recommend that the researchers screen about twice as many individuals than they need to achieve a dyadic recruitment goal on Prolific. Among those who passed screening, dyadic completion of the full study was relatively high (196/267, 73.4%). Finding eligible participants to pass the screening was in reality the more challenging hurdle because it involved both the dyad members completing the screening, including questions for which the responses could have changed between the Prolific prescreening and our screening process (eg, partner no longer uses Prolific). For cohort 2, with only 1 participant to screen and cancer survivorship being a fixed screening response, over-recruitment of the same magnitude was not necessary.

Our second aim was to maximize the cohort 1 sample diversity with respect to sexual orientation, race, and ethnicity. We consider our efforts partially successful. Approximately 24.9% (119/478) of the cohort 1 sample identified with an underrepresented sexual orientation, about 31.8% (152/478) of the sample identified with an underrepresented racial group, and about 4% (19/478) of the sample identified as Hispanic or Latino. From these data, we can conclude that we oversampled underrepresented groups from the available pool on Prolific. However, the proportion of our sample identifying as Hispanic or Latino falls short of the US population estimates (about 19%; [30]). Therefore, in future studies aiming to obtain a sample of more representatives of the US population, we would recommend more targeted recruitment efforts to oversample participants who identify with Hispanic or Latino ethnicity. In addition, researchers aiming to use stratification methods for online panels as a recruitment strategy should consider exclusively opening the survey to targeted recruitment populations at the start, to get a sense of the sampling patterns and efforts required. We also suggest that researchers contact their recruitment platform before implementing stratification efforts to estimate the length of time it might take to reach sampling goals, and to plan for a longer data collection period compared with a nonstratified sampling study.

Although efforts were successful in recruiting a dyadic sample inclusive of those with sexual orientations not well-represented in previous research, it is important to note the limitations associated with Prolific’s sexual orientation prescreening criterion. Specifically, Prolific’s sexual orientation question includes limited response options (ie, “heterosexual,” “homosexual,” “bisexual,” “asexual,” and “other”) which may fail to capture the full range of diverse sexualities present among Prolific’s participant pool at the time of data collection, and the exclusive use of the term “homosexual” may be deemed offensive by some who prefer to identify as gay or lesbian, potentially leading to inaccurate responses or discomfort for the participants.

Our third aim was to recruit a sample of 372 cancer survivors. This goal was achieved; 444 participants completed the time 1 survey, and 375 participants completed the time 2 survey. When we began the study, a total of 731 cancer survivors were eligible; this indicated a high rate of engagement or interest in participation, and low attrition among the cancer survivor cohort. Data collection for cohort 2 took approximately 9 months. Given that the available pool of cancer survivors was small (n=731), it was not feasible to conduct a dyadic study in which 1 partner was a cancer survivor or to prioritize stratified recruitment to maximize sample diversity with respect to race, ethnicity, or sexual orientation. Near the end of data collection for cohort 2, we closed the main survey open to all individuals who met our inclusion criteria and opened a separate survey that was only open to individuals who identified as one of the following ethnicities: African, Black or African American, Caribbean, East Asian, Latino or Hispanic, Middle Eastern, Mixed, Native American or Alaskan Native, South Asian, Other, White or Sephardic Jew, Black or British, White Mexican, Romani or Traveller, or South East Asian (variable “ethnicity”). The survey was open for approximately 4 weeks and 7 participants completed the study. Given that we were nearing the end of our planned data collection period, we decided to close this survey and end data collection at that time. Our recommendation to researchers aiming to recruit underrepresented populations on Prolific is to be mindful of the inclusion criteria they use and how it may further restrict their sample size. In addition, patience is key.

### Challenges and Successes During Study Design and Data Collection

We would like to acknowledge that we had an overall positive experience using Prolific as our recruitment platform. Prolific has a very responsive support team that was able to address questions and issues that arose, from data collection to data cleaning. However, there were some challenges associated with using Prolific with our study design and we learned a few lessons while using the platform that may be useful to other researchers. First, researchers should be aware that Prolific prevents individuals in the same household (according to IP address) from taking the same survey. We realized this when piloting our survey with a small number of dyads for cohort 1 (which we recommend researchers do to minimize data collection errors at a larger scale). This is intentional—the goal is to prevent individuals from taking the same survey twice [25]. However, this feature of Prolific means that researchers must send the members of a dyad 2 separate invitations to enable them to complete the study. We set up 2 studies in Prolific, one for Partner A and one for Partner B, and manually sent separate study invitations to participants by adding them to custom-allow lists. This meant that we had to identify Partner A and Partner B in each dyad, keep them straight throughout data collection, and take the time to manually send surveys to participants using the custom-allow lists. Although this can be done in batches (by setting up surveys so that they are accessible by invitation only), this is time consuming compared with the typical online survey. This poses a limitation to researchers who aim to send surveys to individuals immediately when they become eligible for the study.

It was found that one strength of using Prolific and other online platforms in behavioral research, especially in a COVID-19 pandemic or postpandemic world, is that we can achieve better reach. This was especially crucial for our recruitment of cancer survivors, as this population may have been even more challenging to reach if we had required an in-person component of our study, given their increased health risks during the pandemic. Most recruitment platforms also provide the ability to send participants study reminders—we recommend investigating if this is done automatically and otherwise deciding a priori intervals to send reminders manually. Online survey methods and platforms have become more critical tools than ever before.

Related to the COVID-19 pandemic, there were several challenges with respect to our study design and data collection. The primary challenge was the need to be responsive to pandemic-related changes in the items that we included in our surveys. For instance, as variants evolved and vaccines became more widely available, there were changes in individuals’ risky behaviors (and what was considered risky), their perceptions, etc. This is something that will need to be addressed in all the analyses that use the data from this work. In addition, there were changes in vaccine availability and recommendations across the data collection period (eg, vaccine recommendations for children). Furthermore, there were pandemic-related differences between the United States and the United Kingdom that need to be accounted for, such as governments’ responses to the pandemic and cultural perceptions of the pandemic.

There were also several challenges in collecting dyadic, repeated measures data online. First, managing data collection for dyads is quite complex and requires frequent team meetings for the purpose of decision-making (eg, whether to use data from partial dyads or whether to allow individual partners to continue the study if their partner is not responding). Second, we identified that there is a need to verify that participants are (1) in cohabiting relationships with the other participant they identified on Prolific and (2) are not completing the study for their partner. In other words, there is a need to verify that 2 people are in a dyad but that both members of the dyad completed their respective surveys independently of one another. We recommend that researchers include questions for which the responses should differ for partners (and questions for which the responses should be the same). For instance, partners should generally indicate similar time frames for the start of their relationship and cohabitation, as well as residing in the same county if cohabitating; partners should generally report different specific heights and weights. We also recommend examining the times that participants start and complete their surveys compared with their partners and paying close attention to dyads who complete their surveys at close times. To protect participants’ privacy, we chose to disable the collection of IP address data in Qualtrics, however, this data could potentially be useful for data quality checks, as well. We recommend contacting a recruitment platform directly with questions about data quality. Prolific does have protocols to retain users who provide high-quality data [23].

### Conclusion

With this protocol paper, we aimed to describe the decisions and methodological approaches that were used in the R2C2 study to inform best practices for future online research on dyadic relationships and health. Online data collection methods can enable research teams to obtain cross-sectional or repeated-measures data from participants and their romantic partners; however, obtaining data from underrepresented populations such as cancer survivors and medically underserved populations was a challenge. We encountered several important considerations for designing dyadic health research in particular that have not been previously described in detail. We recommend conducting pilot studies to test the infrastructure of the data collection process—screening at least 2 times the number of individuals that complete data is needed in order to account for the attrition that occurs with this panel-based design, as well as planning dyad-specific data quality checks. For recruiting large samples of underrepresented populations, we recommend allowing for extra time in the data collection timeline and working with the online panel to create realistic expectations for recruitment goals. Relationships are significant to all aspects of life including health, and with valuable tools and sound methods, online research in this area can make worthwhile contributions to further our understanding of this complex association.

## Appendix

Multimedia Appendix 1 Measured constructs and example items.

## Abbreviations

APIMPowerR: Power Analysis for Actor-Partner Interdependence Model
CAPTCHA: Completely Automated Public Turing test to tell Computers and Humans Apart
LGB: lesbian, gay, bisexual, or pansexual
NCI: National Cancer Institute
R2C2 study: relationships, risk perceptions, and cancer-related behaviors during the COVID-19 pandemic study
