# Supplementary material for: Measuring Relationship Influences on Romantic Couples’ Cancer-Related Behaviors During the COVID-19 Pandemic: Protocol for a Longitudinal Online Study of Dyads and Cancer Survivors
Source: JMIR Res Protoc. 2024 Jul 31;13:e48516. doi: 10.2196/48516 (PMC11325112; doi:10.2196/48516)
Supplement: Multimedia Appendix 1 [file resprot_v13i1e48516_app1.docx]

| Construct or  Measure | Total number of items | Example item | Cohort 1 Time 1 | Cohort 1 Time 2 | Cohort 2 Time 1 | Cohort 2 Time 2 |
| --- | --- | --- | --- | --- | --- | --- |
| COVID-19 history and behaviors | 8 | In the previous 7 days, how often have you worn a mask when indoors in public or at a gathering with people who are unvaccinated for COVID-19 or whose vaccination status is unknown? | **×** | **×** | **×** | **×** |
| COVID-19 behavioral intentions | 7 | Over the next month, I plan to avoid going out to bars/pubs or restaurants indoors. | **×** | **×** | **×** | **×** |
| COVID-19 vaccine status and attitudes | 4 | Why did you get a COVID-19 vaccine? | **×** | **×** | **×** | **×** |
| COVID-19 partner pressure | 2 | Has your partner tried to pressure you about the vaccine? | **×** | **×** | **×** | **×** |
| *“*Perceived Stress Scale*”* since the COVID-19 pandemic | 4 | How often have you found that you could not cope with all the things you had to do? | **×** | **×** | **×** | **×** |
| COVID-19 risk perceptions–self | 5 | How likely is it that you will get coronavirus (COVID-19) at some point in the future? | **×** | **×** | **×** | **×** |
| COVID-19 history and risk perceptions–partner | 7 | How worried are you about your partner getting coronavirus (COVID-19) in the future? | **×** | **×** | **×** | **×** |
| Cancer history | 4 | Have you ever been diagnosed as having cancer? | **×** |  | **×** |  |
| Cancer risk perceptions–survivor | 6 | How likely is it that you will develop cancer (either recurrence or a new type of cancer) at some point in the future? |  |  | **×** |  |
| Cancer risk perceptions–no cancer history | 5 | How easy is it for you to imagine developing cancer in the future? | **×** |  |  |  |
| Cancer history and risk perceptions–partner | 6 | If your partner develops cancer, how likely is it that they will die from it? | **×** |  | **×** |  |
| General health–self and partner | 2 | Would you say that in general your health is…Poor, Fair, Good, Very Good, Excellent | **×** | **×** | **×** | **×** |
| Perceptions of COVID-19 cases | 2 | Would you say that the number of coronavirus cases in your area is… (1-5) Trending towards going down…Trending towards going up | **×** | **×** | **×** | **×** |
| Relationship satisfaction | 3 | How satisfied are you with your relationship? | **×** | **×** | **×** | **×** |
| Dyadic stress and coping | 9 | How helpful is your partner when you need support? | **×** | **×** | **×** | **×** |
| Dyadic coping using health risk behaviors | 4 | If my partner has a bad day, I suggest having a beer, glass of wine, or other alcohol beverage to help them feel better. | **×** | **×** | **×** | **×** |
| Dyadic health management | 2 | Managing each person’s health is really the responsibility of both partners in a relationship. | **×** |  | **×** |  |
| Health behaviors with partner | 9 | During the past 7 days, how often did you exercise together with your partner | **×** | **×** | **×** | **×** |
| Household and life events during COVID-19 | 9 | Do you do more childcare now, as compared with before the coronavirus (COVID-19) pandemic? | **×** |  | **×** |  |
| “Everyday Discrimination Scale” | 6 | In your day-to-day life how often… You are treated with less courtesy or respect than other people. | **×** | **×** | **×** | **×** |
| Emotion regulation | 4 | I control my emotions by changing the way I think about the situation I am in. | **×** |  | **×** |  |
| Attention check | 1 | Please select “strongly agree.” | **×** | **×** | **×** | **×** |
| Depressive symptoms | 4 | In the past 7 days… I felt hopeless. | **×** | **×** | **×** | **×** |
| Post-traumatic stress symptoms | 6 | In the past month, have you... Had nightmares about the event(s) or thought about the event(s) when you did not want to? | **×** |  | **×** |  |
| Coping with substances – self and partner | 4 | Do you have a desire to drink alcohol when you are upset (e.g., depressed, discouraged, lonely, or anxious)? | **×** | **×** | **×** | **×** |
| Health behavioral intentions | 2 | I plan to eat a healthy diet over the next month. | **×** |  | **×** |  |
| COVID-19 isolation | 2 | To what extent did you hold back from expressing your feelings during the last conversation you had with your partner related to coronavirus (COVID-19)? | **×** | **×** | **×** | **×** |
| Physical activity | 7 | During the LAST 7 DAYS, on how many days did you WALK for at least 10 minutes at a time? | **×** | **×** | **×** | **×** |
| Sedentary behavior | 2 | During the LAST 7 DAYS, how much time did you spend SITTING on a WEEKDAY? | **×** | **×** | **×** | **×** |
| Sleep | 4 | During the last 7 days, how would you rate your sleep quality overall? | **×** | **×** | **×** | **×** |
| Alcohol | 5 | During the past seven days, on how many days did you have at least one drink of any alcoholic beverage? | **×** | **×** | **×** | **×** |
| Tobacco | 7 | Have you smoked at least 100 cigarettes in your entire life? | **×** | **×** | **×** | **×** |
| Dietary intake | 15 | During the past 7 days, how often did you eat fruit? Do not include juices. | **×** | **×** | **×** | **×** |
| Height/weight | 2 | What is your weight without shoes? | **×** | **×** | **×** | **×** |
| Relationship information | 3 | When did your current romantic relationship begin? | **×** |  | **×** |  |
| Household information | 4 | Do any children under the age of 18 live in your household? | **×** |  | **×** |  |
| Demographics | 14 | What best describes your current employment status? | **×** |  | **×** |  |
